# Chasing Happily Ever After: Psychometric Development and Nomological Validation of the Rescue Fantasy Beliefs Scale

**DOI:** 10.3390/bs16071113

**Published:** 2026-07-03

**Authors:** Stephen Bok, James Shum, Maria Lee

**Affiliations:** 1 College of Business and Economics, California State University, East Bay, 25800 Carlos Bee Blvd, Hayward, CA 94542, USA; 2 School of Accounting, Golden Gate University, 536 Mission St, San Francisco, CA 94105, USA; 3 Department of Urban Planning and Public Policy, University of California, Irvine, Irvine, CA 92697, USA

**Keywords:** attachment theory, relational arrival fallacy, rescue fantasy beliefs, expected relational disappointment, current relational satisfaction, shopping addiction

## Abstract

Based on attachment theory, individuals develop relational schemas that shape cognitive-emotional social relationship expectations (e.g., others are a source of safety). Social relationships (e.g., intimate relationships or close friendships) are a source of long-term happiness. However, expectations that they will save someone from life’s challenges are a common fallacy (e.g., a shining prince/princess bringing lifelong happiness). This places illusionary expectations on others to not disappoint despite normal behavioral realities (e.g., relational misunderstandings and conflict). This project psychometrically developed the rescue fantasy beliefs (RFB) and expected relational disappointment (ERD) scales. Analysis of the scales demonstrated satisfactory reliability, discriminant validity, and convergent validity. Serial mediation analysis demonstrated that higher RFB is associated with higher shopping addiction. ERD and current relational satisfaction sequentially mediated this relationship. The results demonstrated a serial connection between RFB and lower ERD. This serial illusionary expectation gap in others is associated with lower current relational satisfaction and higher shopping addiction. Addictive shopping can function as a compensatory coping strategy to unmet social needs. Business marketing implications discuss how new offerings can encourage meaningful in-person social connections to better address underlying needs (for those with greater RFB).

## 1. Introduction

Rescue fantasies reflect savior expectations in others to protect, give care, or provide emotional support (Esman, 1987; Gillman, 1992). Such relationships function as idealized solutions (e.g., savior idealization) to personal distress (Malawista, 2004). A seemingly nurturing relationship can form a savior-like attachment bond, seeking to relieve emotional distress (Neumann & Gamble, 1995). Prior research described how personal traumas have spurred many individuals to pursue therapeutic careers to rescue others, which can go beyond professionally helping clients because of deeply felt emotions (McWilliams, 1984). While studied in a therapeutic setting, the prevalence of unhealthy developmental attachments and commonality of these beliefs extend unconsciously to the general population (Athanasiadou-Lewis, 2019; Bader, 2003, p. 48). For example, the pursuit of intimate partners often resembles archetypal plotlines of movie fantasies (e.g., “If only I got married, then I will live happily ever after”) (Nelson, 2024; Wexman, 1993; White, 1989). At present, there lacks a rescue fantasy beliefs (RFB) scale to operationalize the belief that close relationships will save someone from loneliness, hardship, and low self-worth with lasting support. A psychometrically validated measure would allow researchers to study how this phenomenon affects perceptions of relationship quality and outcomes when expectations are unmet.

At present, there lacks a validated measure to capture RFB and its subsequent relational letdown [i.e., expected relational disappointment (ERD)]. When society widely accepts this happily ever after narrative (as portrayed in movies), it suggests RFB reflects many consumers’ belief systems (Valkenburg & Peter, 2013). The purpose of this study is to psychometrically develop the novel RFB scale and quantify serial relationships that accompany this phenomenon. RFB hypothetically sets unrealistically high expectations in relationships that likely will fall short of lasting happiness that eliminates lifelong feelings of loneliness and relational distress. RFB is a form of future aspirational thinking in the transformation of one’s life (e.g., buying a more expensive wedding ring, imagining that it will help forge happiness into the relationship forever). This places aspirational meaning into decisions like heavily investing in certain relationships and objects bought. RFB extends beyond romantic relationships and can apply to how people can view close connections (e.g., expectations of a best friend). Someone who is asexual may also lack romantic interest in others but still place RFB on others. RFB can apply to individuals regardless of romantic interests and relationship status, which opens opportunities to advance consumer behavior research in studying individuals with this mindset.

The present study pursues two integrated objectives. First, this study develops and validates a psychometric scale measuring rescue fantasy beliefs (i.e., idealized unrealistic expectations in others to resolve loneliness, life struggles, and deficits in self-worth). Second, this study examines the serial relational mechanisms through which rescue fantasy beliefs relate to unmet social needs and affect shopping addiction (i.e., compulsive and repetitive purchasing motivated by unmet psychological needs versus utilitarian product value) (L. Guo et al., 2023; Niedermoser et al., 2021). Grounded in attachment theory, the hypothesized model proposes that RFB operate through expected relational disappointment (ERD) (i.e., anticipation that romantic relationships will fail to meet idealized expectations) and current relational satisfaction (i.e., the degree to which an individual’s present relationship fulfills their relational needs) in sequence (Gander et al., 2025). Collectively, these objectives address a theoretical gap between attachment-based relational cognition and compensatory consumer behavior research. The goal of the current project is to develop the rescue fantasy beliefs (RFB) scale and perform nomological validation by testing the theoretically supported hypothesized relationships.

## 2. Literature Review and Hypothesis Development

### 2.1. Attachment Theory and the Arrival Fallacy

Attachment theory explains that, across one’s lifespan, individuals form relational schemas that guide expectations, emotional regulation, and coping strategies (Bowlby, 1969; Mikulincer & Shaver, 2010). These relational schemas influence how individuals anticipate relational fulfillment (J. Cassidy & Shaver, 1999). They influence how individuals respond to perceived successes or disappointments in close relationships. Secure attachment is associated with greater emotional regulation and relational satisfaction, whereas insecure attachment is linked to heightened reliance on external sources to meet unmet relational and emotional needs (Mikulincer & Shaver, 2010).

The arrival fallacy refers to the mistaken belief that achieving a desired relational or life outcome will produce enduring happiness and emotional fulfillment (Ben-Shahar, 2007). Although the term “arrival fallacy” originates from positive psychology, its underlying mechanisms are strongly supported by empirical research on affective forecasting errors and hedonic adaptation (Diener et al., 2006; D. T. Gilbert et al., 1998). This suggests that individuals systematically overestimate the long-term emotional benefits of future goal attainment. Upon arriving, there is a letdown from the absence of permanency in desired outcomes (Dorr, 2017; Masta, 2025). Colloquially, people express the arrival fallacy when they say, “If I win the lottery, my life will be better because I will no longer have any debt.” Similarly, rescue fantasy believers say, “If only I had a boyfriend/girlfriend, my life will be better because I will no longer be lonely.” Hypothetically, this new partner will do activities together (e.g., work out at the gym), improve their health, and lift them to a new state of wellbeing. This is a relational form of the arrival fallacy, where a destination (i.e., in a relationship) will result in a desired outcome (Diener et al., 2006; Pavot & Diener, 2013). This places savior expectations in others to resolve common human challenges that most struggle to overcome throughout their lifetime. The attachment theory framework aligns well in explaining a relational form of the arrival fallacy [i.e., rescue fantasy beliefs (RFB)].

Insecurely attached individuals are more likely to link emotional security and self-worth to anticipated relational outcomes (or idealized relational fantasies) (Mikulincer & Shaver, 2007). Mikulincer and Shaver (2007) studied how such group counseling participants would project idealized qualities onto peer group members and therapists. When these high relational expectations are not met (e.g., harmonious, perfect, and completely supportive), they can experience disappointment. This suggests that RFB are part of a cognitive process that can sequentially shape how individuals anticipate disappointment and evaluate satisfaction in their close relationships (Bowlby, 1969; Gander et al., 2025). Thereby, we contend that the construction of the RFB measure is closely linked to perceived relational outcomes. Additionally, the literature has yet to examine how idealized relational belief systems (versus individual personality traits alone) contribute to compensatory consumer behavior when relationships fail to meet unrealistic expectations. There is a gap in understanding how unfulfilled relational needs can result in compensatory behaviors (e.g., shopping addiction). Taking into account complex relational factors, we investigate factors that influence shopping addiction through serial relational mechanisms. This is why we conduct psychometric development of the RFB scale and nomological validation through hypothesis testing.

### 2.2. Rescue Fantasy Beliefs, Self-Regulation, and Compensatory Behaviors

Within the attachment theory framework, RFB functions as an attachment-based cognitive representation of others. RFB are theoretically distinct from adjacent constructs. While insecure attachment reflects a broad dispositional relational orientation rooted in early caregiving (i.e., generalized anxiety or avoidance across relationships), RFB captures the belief that others will rescue them from emotional and self-worth deficits similar to heroic romantic media depictions (Kretz, 2024; Kretz & Hefner, 2024). While positive illusions idealize a partner’s traits and qualities internally to sustain relational motivation, RFB idealizes external functions of the relationship to improve internal deficits (Murray & Holmes, 1997). While relational optimism reflects generalized positive relational expectations, RFB is deficit-motivated (Gander et al., 2025). While relational dependency reflects behavioral over-reliance on an existing partner, RFB operates prospectively in the absence of a relationship (i.e., promotes partner seeking rather than partner reliance) (Bornstein, 2012). Lastly, while contingent self-worth refers to self-esteem that fluctuates based on relational acceptance or rejection, RFB precedes relational experiences and motivates pursuing relationships before outcomes materialize (Crocker & Wolfe, 2001). RFB is, therefore, distinct from existing constructs in cognitive processing, prospective orientation, and deficit motivation.

RFB idealizes relational outcomes to resolve underlying emotional insecurity and relational needs. Such RFB can temporarily reduce perceived ERD with symbolic security and hope for future fulfillment (even if objective relational change is lacking) (Mikulincer & Shaver, 2010). Similar to the arrival fallacy, outcomes are fleeting and often disappointing because satisfaction from goal attainment shifts soon after obtaining the aspirational goal (Diener et al., 2006, p. 206; D. T. Gilbert et al., 1998). Emotional relief from an anticipated arrival to an ideal relationship is unlikely to have sustained fulfillment. Happiness tends to be in experiencing the present rather than a fantasied future (Ben-Shahar, 2007; Pavot & Diener, 2013). The combination of high expectations and practice of extending goals to new limits can create a cycle of not feeling good enough (Diener et al., 2006). We contend that RFB represents an insecurely attached relational schema. Insecurely attached individuals form psychological defense systems to mask feelings of unmet social needs (Simpson & Rholes, 2017). Unmet social needs can be from past developmental relationships or current relationships based on how one approaches them (e.g., anticipatory withdrawal that sabotages meaningful relationships before they develop) (Baumeister & Leary, 1995). Thereby, RFB can increase susceptibility to relational distress, because internal wellbeing is partially outsourced to others (as opposed to self-regulated).

RFB are a future-oriented belief system that can aid the study of marketing outcomes. Attachment theorists explain how insecure attachment individuals will exhibit compensatory behaviors to cope with distress and restore a sense of security (Mikulincer et al., 2003). Addictive shopping behaviors are considered a maladaptive approach to soothing and regulating emotions (Hartston, 2012). Qualitative research found a positive association between insecure attachment styles and compulsive shopping behaviors (Topino et al., 2022). Insecurely attached individuals will turn towards material objects and money as sources of self-worth and emotional security (compared to securely attached individuals) (Mikulincer & Shaver, 2008). A study of individuals who experienced attachment trauma found that they were likely to participate in compensatory behaviors that aimed to restore or transform one’s state (Caldwell & Henry, 2017). They seek a state of confidence, equilibrium, and a sense of control over the trauma. Conversely, satisfying social relationships are associated with reduced reliance on compensatory consumption because secure relational bonds fulfilled attachment needs (Mikulincer & Shaver, 2010).

A lack of emotional self-regulatory control typically spurs alternative coping behaviors. Low self-control and high neuroticism correlated with higher shopping addiction (Andreassen, 2015). Andreassen (2015) explains shopping addiction is a learned behavior in late adolescence or early adulthood that promotes distress. It is maladaptive and can impair one’s wellbeing through preoccupation. Furthermore, researchers found that negative emotions (e.g., stress) influence compulsive e-commerce buying, which provides temporary emotional relief (Chaudhary et al., 2025). Consumers can feel emotional stability when engaging in addictive shopping behaviors (T. Cassidy & Adair, 2021). Despite long-term consequences from excessive buying, it is often used to manage negative emotions and psychological distress. The act of shopping provides a psychological sense of control that can help cope with relational distress.

**H1.**  *Rescue fantasy beliefs (RFB) will be associated with higher shopping addiction.*

In the absence of direct research studying RFB, we draw from intimate partner studies of positive illusions (i.e., idealization). Positive illusions include an idealistic view of partners, inflated perceived control over the relationship’s future, and unrealistic optimism in the relationship (Murray & Holmes, 1997; Niehuis et al., 2011). Positive illusions project a positive outlook on relationships and partners. This means less anticipatory negative emotions, like distress and disappointment. This is similar to moviegoers’ positive anticipation before visiting a theater to see a highly acclaimed romantic film (not expecting to be disappointed). With regard to relationships, this means not expecting disappointment in others (even though both positive and negative emotions are a normal part of interpersonal relationships) (Mortensen, 2006). Attachment theorists explained how idealized beliefs about romantic relationships form relational schemas that can minimize anticipated relational failures (i.e., reduce ERD) (Stackert & Bursik, 2003).

Based on attachment theory, internal relational schemas influence how individuals interpret closeness and disappointment in relationships (Overall et al., 2013). This can be projected onto anticipated relational outcomes. Prior research found that secure attachment styles are associated with greater perceived partner responsiveness, greater trust, and lower relational distress (Arriaga et al., 2018). Meanwhile, insecure attachment styles are associated with greater sensitivity to rejection and unmet expectations (Campbell et al., 2010). Nurturing partner support can buffer stressful or threatening events that activate felt insecurity (Simpson & Overall, 2014). Emotional and behavioral regulatory actions can soothe worries experienced by insecurely attached individuals. This suggests that individuals can adapt their relational schemas by thinking differently to feel better about their social wellbeing. For example, in parasocial relationships (i.e., believing someone is in an intimate relationship with a public figure), someone can pour their emotions into an idealized celebrity and never be disappointed by their normal human behaviors (Hartmann, 2016; Lotun et al., 2024). This creates perceived relational fulfillment and safe attachment under the disguise of an illusionary narrative (Degen, 2023). Degen (2023) explains that parasocial relationships are cognitive constructs that overly attribute reciprocity to give a sense of connection. For instance, an idolized singer saying to a concert audience “I love you all” can be misinterpreted as a personal expression of intimate love. We contend RFB projects savior fantasies onto others to fill gaps in existing relational wellbeing. By projecting RFB onto others, it cognitively protects oneself from the common realities of relational disappointment (i.e., belief that social relationships will fail to resolve life challenges, loneliness, and distress) (Miller-Day & Lee, 2001; Vangelisti et al., 2002). It acts as a preemptive cognitive buffer. For example, if someone focuses on how their spouse always comes through in picking up the kids after work, inadequacies can become downplayed (e.g., poor listening). By projecting RFB onto others, disappointment from unmet expectations can be cognitively downplayed (despite objective realities). This projects positive attributes onto future relationship outcomes.

Moreover, insecure attachment poses a risk factor for developing maladaptive coping patterns as a secondary regulatory strategy (Fraley, 2019). Fraley (2019) explains that when stress activates one’s attachment-related system, insecure individuals tend to use hyperactivating or deactivating coping strategies. This alternatively externalizes primary direct support seeking. Meanwhile, securely attached individuals can more constructively manage disappointment in intimate relationships (Feeney, 1999). Relational distress in close relationships can be mitigated with perceived responsiveness and felt security (Reis et al., 2004). Therefore, RFB may reduce ERD by cognitively misattributing dependable support. For example, fictional princes or princesses usually always save the kingdom despite adversity; believing otherwise would contradict this archetypal storyline. We contend that this illusionary narrative is applied to others in real life. This acts as a preemptive defense from negative relational emotions. This is like Cinderella (in the fairytale) escaping negative familial conditions to be with a loving prince that perceives her as beautiful without needing any change (Panttaja, 1993). Following the illusionary illogical thinking of this relational arrival fallacy, how could there be anything wrong with this savior figure? RFB projects savior attributes onto others to conceal relational distress from typical interpersonal conflicts and challenges.

**H2.**  *Rescue fantasy beliefs will be associated with lower expected relational disappointment (ERD).*

Consistent with attachment theory, ERD reflects unmet relational needs that activate compensatory behaviors, such as shopping as an emotional regulatory strategy (Gillath et al., 2016). For instance, insecurely attached individuals tend to have more difficulty regulating personal emotional experiences (Shaver & Mikulincer, 2007). Emotional regulation includes soothing oneself when stressed or after experiencing negative events (P. Gilbert et al., 2008; Rolston & Lloyd-Richardson, 2017). Anxiously attached individuals tend to overreact or ruminate when distressed (Malik et al., 2015; Mikulincer & Shaver, 2019). Avoidantly attached individuals tend to suppress or withdraw when distressed. Hyperactivating or deactivating emotions add ongoing relational tensions that tend to lower personal wellbeing (Karreman & Vingerhoets, 2012). Greater relational distress increases reliance on external emotional regulatory strategies (Mikulincer & Shaver, 2010). Insecurely attached individuals tend to experience greater relational tension and emotional dysregulation (Malik et al., 2015; Mikulincer & Shaver, 2019). Shopping is one method individuals use to cope with emotional distress (Atalay & Meloy, 2011). Compulsive shopping was studied to be a psychological tool to regulate emotions and improve self-worth (Dittmar, 2005b). Consumers may use marketplace attachments to substitute for relational deficits and to feel symbolic security (Clark et al., 2017). For example, consumers develop brand attachments when a brand elicits strong positive affect and consumers integrate the brand into one’s self-concept (Park et al., 2010). Brands, shopping, and material goods can soothe emotional dysregulation (even though they may not address underlying sources of distress).

Combined, attachment theory and the arrival fallacy explain why RFB may reduce ERD while increasing vulnerability to shopping addiction. Parallel to the arrival fallacy, RFB may temporarily buffer relational disappointment with expectations of future fulfillment (Diener et al., 2006; D. T. Gilbert et al., 1998). However, this elusive illusionary future often lacks sustained emotional security, which can drive compensatory shopping to cope (Akın, 2025). For example, just because someone gets married with heartfelt vows does not necessarily guarantee long-term happiness (Lyubomirsky, 2014). If the relationship declines, individuals often use objects to try to repair (e.g., flowers) or cope with dissatisfaction (e.g., golf clubs to support a hobby to escape) (Komter, 2007). Thereby, shopping can act as an alternative outlet to cope with relational stress and disappointment. Most shopping is an autonomously rewarding activity without common complications that accompany many social relationships (e.g., disagreements and last-minute cancellations) (Li et al., 2023). Addictive shopping preoccupies one’s mind by deeply engaging with the buyer behavior process (e.g., timing purchases with sales and excessive comparison of product features) (Niedermoser et al., 2021). This can shift time away from ruminating on disappointment and unmet expectations. Compulsive shopping can distract someone from feeling negative relational emotions (even though they are a normal part of interpersonal relationships) (Saraneva & Sääksjärvi, 2008). For example, after feeling unseen at a social gathering, someone can shop for a new gadget or attire to internally feel worthy or to impress next time. Not everyone is a fantasized prince or princess that will bring joy into one’s life, absent from conflict. Negative emotions (e.g., disappointment) are a normal part of relationships (Behrendt & Ben-Ari, 2012). Expecting only (or mostly) positive emotions to come from close relationships contradicts reality (Oatley & Johnson-Laird, 2011). This closely aligns with RFB as a preemptive emotional regulatory strategy to cope with relational disappointment. Hence, when disappointment ensues from savior expectations, shopping addiction becomes an alternative means to cope with unmet relational needs. We expect higher ERD to associate with greater shopping addiction.

**H3.**  *Expected relational disappointment will be associated with higher shopping addiction.*

Attachment theorists explain how idealized relational schemas create relational dissatisfaction when reality misaligns with romanticized expectations (Gander et al., 2025). RFB can serve as a criterion to compare current relationships. Attachment theorists explained how developmental caregiving experiences shape relational schemas that influence later relational expectations in closeness, support, and responsiveness (Fraley & Shaver, 2008; Pietromonaco & Barrett, 1997). Relational schemas become scripts or rules for behaving and thinking (Bowlby, 1969; Fraley & Shaver, 2008). For example, early childhood feelings of self-worth are often derived from positive reinforcement from parental figures. When it is not provided by others, a child can develop a narrative of unworthiness because internal beliefs of self-worth are not matched by external validation (Tayler, 2015). A pattern of a child’s actions and parental responses develops beliefs and learned behaviors (about others and themselves) (Murphey, 1992). Relational schemas are shaped through interactions and responses. Furthermore, meta-analysis of attachment and romantic relationship outcome studies found insecure attachment to be negatively associated with relationship quality and satisfaction (Hadden et al., 2014). Avoidantly attached individuals react negatively when partner support is either insufficient or overly intrusive (Girme et al., 2015), whereas moderate and autonomous-respecting social support was associated with higher relationship satisfaction. Idealized relational schemas (i.e., RFB) distort how individuals evaluate their current relationship because reality appears unsatisfactory compared to idealization. This follows the comparative evaluation process.

Humans are flawed and often let down others (e.g., show up late or forget to perform promises) (Hallinan, 2009). Setting high expectations increases this gap between exalted belief in others and what occurs (Lemay & Venaglia, 2016). This creates greater odds of perceived dissatisfaction because of an unmet list of unsatisfied expectations (Sullivan & Schwebel, 1995). For instance, seeking someone passionate about their career, physically fit, without ailments, stress-free, beautiful, and ready to salvage a partner from debt is a lofty romanticized standard. It is human nature to conserve energy through procrastination and sedentary lifestyles (Prapavessis et al., 2015; Rollo et al., 2016). Individuals often show up just on time and with their own list of problems (Ariely & Wertenbroch, 2002). Savior expectations deny common realities. While there are excellent potential partners, RFB projects unrealistic expectations onto others, perpetuated by idealized fantasy stories (Wexman, 1993; White, 1989). Charming princes or princesses are uncommon, even though the mythical belief that this random person will appear in someone’s life is common (Lucchi Basili & Sacco, 2018). Life is complicated, and people often show up each day trying to figure out what to do; they are not looking to resolve other people’s problems (Norman, 2016). The illusionary RFB in others is a psychological mechanism to cope with one’s struggles. By externalizing help from others, it provides a false sense of security that can relieve negative feelings (e.g., feeling alone) (Lawrence & Valsiner, 2003). When compared to one’s current relationship, it can sink perceived satisfaction.

Relational schemas influence how interpersonal interactions are perceived in their relative support and thereby the evaluation of relationship satisfaction (Pietromonaco & Beck, 2019). Rescue fantasies idealize others to provide care, protection, and emotional salvation (Esman, 1987; Gillman, 1992). Idealized expectations can generate disillusionment in intimate relationships from discrepancies in imagined and actual experiences (Niehuis et al., 2011). When individuals trust a partner to be reliable and benevolent, this reduces emotional distress (Simpson, 2007). Partners in such secure relationships are more likely to be vulnerable, cooperate, and experience relationship satisfaction. Contrarily, when a partner is less vulnerable and cooperative, this can decrease relationship satisfaction. Possessing the right amount of these characteristics illustrates unrealistic expectations that, when matched up against one’s current relationship, can be dissatisfying.

Relationship beliefs shape how individuals interpret and respond to conflict among couples (Knee et al., 2004). In their diary study of couples, Knee et al. (2004) found that a destiny belief (i.e., relationship meant to be) attributed conflicts as evidence of a compatibility mismatch. Parasocial relationships personify illusionary relational beliefs in others to fulfill social needs (Lotun et al., 2024). Strong parasocial relationships (in adolescence) are associated with greater idealized romantic beliefs in real-life romantic partners (i.e., unrealistic expectations of love and relationships) (Tukachinsky & Dorros, 2018). This is associated with lower real-life relationship satisfaction and lower favorability towards their current partner. This indicated an establishment of unrealistic expectations that real relationships cannot meet. Parasocial relationships (i.e., fantasized cognitive relationships) can function as symbolic substitutes to real relationships (Derrick et al., 2008). Derrick et al. (2008) found that these fantasized relationships helped to improve one’s self-esteem and self-concept not fulfilled by real relationships. While parasocial relationships can reduce loneliness, unrealistic expectations can carry over into real-life relations (Liebers & Schramm, 2019). RFB represents unrealistic expectations in others that can strain relationships (e.g., expecting a partner to always pay restaurant checks). Unrealistic expectations of partners can decrease relationship satisfaction because partners may not live up to these ideals and feel devalued.

**H4.**  *Rescue fantasy beliefs will be associated with lower current relational satisfaction.*

Attachment theorists studied how secure relational satisfaction can reduce the need for compensatory coping mechanisms (e.g., compulsive shopping) by fulfilling the fundamental safety and social needs (Lemay & Venaglia, 2016). Close adult relationships can become key attachment figures that help regulate emotions and maintain psychological security (Hazan & Shaver, 2017). However, unmet attachment needs foster compensatory psychological processes to fill felt relational deficits (Gillath et al., 2016). Gillath et al. (2016) explained that prior relational memories can influence placement of attention as a preemptive defense mechanism from psychological harm. For example, avoiding parents can shift attention away from experiencing past and current pain. Individuals who feel understood and cared for by partners experience less stress and better emotional regulation (Selcuk & Ong, 2013). Meanwhile, poor partner responsiveness and unmet emotional needs promote seeking alternative coping mechanisms. Close relationships serve as safe outlets to cope with threats and negative emotions (Collins & Feeney, 2004), whereas in their absence, individuals pursue maladaptive coping strategies. For instance, in the absence of socializing, an individual may engage in online shopping to distract from feeling lonely. Material consumption has been used to compensate for unmet psychological and relational needs (Mandel et al., 2017). By engaging in shopping behaviors, it can relieve deficiencies in interpersonal fulfillment.

Poorer interpersonal functioning patterns are associated with greater online compulsive buying behaviors (Topino et al., 2022). Poor interpersonal environments can pose a risk factor for maladaptive consumption behaviors. Individuals who engage in problematic shopping behaviors tend to use buying to regulate negative emotions and identity-related distress (Müller et al., 2021). For example, consumer brand attachments offer psychological benefits, such as a sense of community and shared identity (Park et al., 2010; Thomson et al., 2005). Lower relationship satisfaction and greater loneliness were linked with greater compulsive buying tendencies (T. Cassidy & Adair, 2021). This suggests that shopping serves as a coping strategy for unmet relational needs. Thereby, we contend that satisfying social relationships reduce the need to regulate emotions through compensatory shopping behaviors. Higher levels of current relational satisfaction should associate with lower shopping addiction.

**H5.**  *Current relational satisfaction will be associated with lower shopping addiction.*

Attachment-related relational schemas shape expectations in partners’ responsiveness and their ability to meet emotional needs within the relationship (Collins & Feeney, 2004). For example, believing a partner cares about listening will influence how they interpret a partner’s behaviors to fit this narrative. Relational schemas shape how individuals interpret others’ behaviors and relationship experiences (Mikulincer et al., 2014). Interactions are evaluated to the degree they meet established expectations. Mikulincer et al. (2014) found that self-esteem threat and mental depletion negatively affected romantic partner responsiveness in independently rated video recordings, whereas priming security among partners facilitated responsiveness. Thereby, emotional self-regulation can help ground relationships to mutually provide support. For example, securely attached individuals exhibited healthier relational resources and self-regulation. This was associated with less online compulsive shopping (Topino et al., 2022).

Meanwhile, RFB reflect idealized romantic beliefs that become expectations in others (Fernández et al., 2023). These beliefs create idealized expectations about how relationships should function, despite objective realities (e.g., value differences and individual needs). Expecting total fusion with intimate partners (i.e., “you complete me”) and expecting love to solve all personal/relationship problems contribute to unhealthy relationship dynamics. For instance, love alone will not resolve strong disagreements and misalignment in goals. One who values financial planning versus a partner who impulsively spends on credit requires more than felt positive emotions. The belief that someone is their life partner is not enough for a healthy long-term relationship. With high relational idealization, real partners will likely struggle to meet imagined standards (Knee et al., 2001). So even though RFB may reduce anticipated disappointment in relationships, high relational expectations can have downstream consequences (e.g., dissatisfaction from unmet expectations).

Discrepancies between objective realities and ideals can create relational distress and negative affect (Baucom et al., 1996). For example, an individual who believes that their partner should automatically understand their feelings without being told and provide needed support is unrealistic. In reality, clear communication is needed. Discrepancies can also decrease relationship satisfaction if there is a focus on shortcomings (Knee et al., 2001; Simpson et al., 2007). Expected relational disappointment is a sign of unmet relational needs, which can reduce relational satisfaction from weakened emotional security and lower perceived partner responsiveness (Overall & Simpson, 2015). Relationship quality is evaluated by the degree relational expectations and emotional needs are fulfilled (e.g., responsiveness) (Reis & Gable, 2015). Positive illusions in relationships can prevent addressing relationship problems by believing issues will resolve themselves (e.g., “love will solve all”) (McNulty & Fincham, 2012). Unmet emotional needs juxtaposed to idealized expectations contribute to dissatisfaction.

Furthermore, lower relationship satisfaction can increase odds of developing maladaptive coping behaviors to fulfill unmet social needs (Pietromonaco & Beck, 2019). Emotional distress and inadequate emotional regulation are linked to problematic buying behaviors, which function as a coping mechanism to unmet psychological needs (Black, 2001). Consumers partake in retail therapy to cope and regulate negative emotions (e.g., sadness and distress) (Rick et al., 2014). Rick et al. (2014) explain that emotions have lingering or residual effects that last beyond an immediate experience. Their two experiments showed shopping restores a sense of personal control.

Additionally, compulsive shopping preoccupies consumers’ minds from facing negative emotions (Nagel & Sârghie, 2023). For example, instead of sitting with negative emotions of feeling alone, consumers are strolling around a mall browsing new seasonally inspired products. Shopping addiction is described as senseless behavior (i.e., low metacognitive awareness) where items bought are not needed and spending is detrimental to one’s economic wellbeing (Liu et al., 2022). Addictive shopping behaviors become thoughtless (motivated by underlying unmet needs) (Shekhawat & Kaur, 2025). Contrarily, satisfying social relationships reduce the need to engage in compensatory consumption because of fulfilling interpersonal support and connection (Atalay & Meloy, 2011; Mendini & Furchheim, 2025).

Therefore, prior research suggests that RFB indirectly influences shopping addiction through ERD and current relational satisfaction. While relational optimism and transactions are a part of relationships, RFB is a unique facet of relational expectations. Idealized social expectations can increase the gap between imagined relationships and objective realities, which influences relational dissatisfaction. ERD will likely lessen current relational satisfaction. This lower relational satisfaction influences self-regulation by increasing compulsive buying behaviors. Hence, RFB are posited to influence shopping addiction through a sequential mediation process through ERD and current relational satisfaction.

**H6.**  *Rescue fantasy beliefs will influence shopping addiction through a sequential mediation process. Rescue fantasy beliefs will be associated with reduced expected relational disappointment. Expected relational disappointment will be associated with reduced current relational satisfaction. Current relational satisfaction will be associated with reduced shopping addiction. This will yield significant indirect effects through expected relational disappointment and current relational satisfaction in sequence.*

In summary, attachment theory establishes that the quality of developmental relationships shapes relational schemas that persist into adulthood, which affect relational expectations and behaviors (Stackert & Bursik, 2003). Rescue fantasy beliefs are idealized, unrealistic expectations about romantic outcomes constructed over time and often disconnected from realistic relational dynamics (Gander et al., 2025). RFB is anticipated to activate compensatory consumption when unrealistic relational needs go unmet (H1) (Niedermoser et al., 2021). These same rescue fantasy beliefs are predicted to reduce expected relational disappointment, as fantasy-based internal working models shield individuals from anticipating relational failure (H2) (Murray et al., 2011). Yet, when expected relational disappointment increases, individuals seek emotional substitutes for unmet relational needs (e.g., shopping addiction) (H3) (Gillath et al., 2008). Furthermore, RFB is anticipated to reduce current relational satisfaction, as idealized expectations create a persistent gap with relational realities (H4) (Vannier & O’Sullivan, 2018). In turn, higher current relational satisfaction reduces shopping addiction by fulfilling fundamental attachment needs for safety and connection (H5) (Lemay & Venaglia, 2016). We contend that RFB operates through additive relational deficits in ERD and relational dissatisfaction, which promote compensatory shopping addiction (H6) (L. Guo et al., 2023).

**Figure 1:**
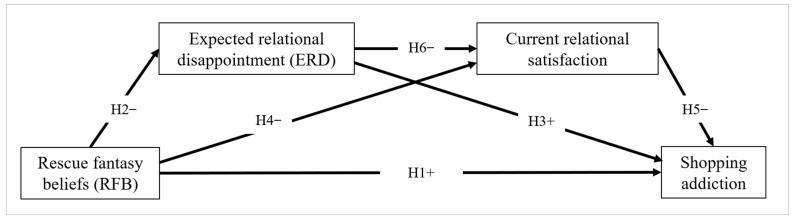
Hypothesized serial mediation modeled effects on shopping addiction.

RFB is a relational construct derived from attachment theory and the arrival fallacy. The hypothesized model of relationships follows the arrival fallacy illogical thinking. A key aspect of the arrival fallacy is a subsequent destination realization that the desired outcome was not met (Dorr, 2017). This brings about a degree of dissatisfaction, emptiness, and confusion because of unmet aspirations of a lasting change (Masta, 2025). This can diminish future motivation because the expected reward is often short-lived. Thereby, when relational aspirations fall short of expected outcomes, alternative behaviors (e.g., addictive shopping) assist self-regulation of unmet needs. We empirically test the serial link between RFB, ERD, current relational satisfaction, and shopping addiction. Figure 1 illustrates the hypothesized serial mediation model.

## 3. Methodology

### Overview of Studies

The project comprised two integrated stages. Stage 1 developed and validated the RFB and ERD through psychometric analysis. Stage 2 compared psychometric properties of the development sample with an independent validation sample. Stage 3 examined nomological validity through hypothesis testing of expected variable relationships.

In stage one, standard scale development procedures using two studies were followed to form the rescue fantasy beliefs and expected relational disappointment scales (Kyriazos & Stalikas, 2018; L. S. Lambert & Newman, 2023; Zhou, 2019). The researchers formulated and refined scale items. Statistical analysis informed the selection of items. The researchers assessed reliability, convergent validity, and discriminant validity. This included data collection of a large sample followed by retesting a portion of the sample for test–retest reliability (Carpenter, 2018; Worthington & Whittaker, 2006). In stage 2, an independent convenience sample was collected, and psychometric values were compared with the development sample. In stage 3, predictive validity (i.e., serial mediation) of the proposed hypotheses was conducted (Hinkin, 2005). Predictive validity was conducted to assess theoretically supported hypothesized relationships between variables and individual differences (Becker et al., 2013; Lim, 2024). This tested the utilitarian value of the measures to study associated variable relationships.

## 4. Stage 1: Psychometric Development

Stage 1 followed a three-phase psychometric development process: item generation (construction of items), item reduction (inter-item correlations and reduction), and final factor structure (factor loadings and descriptive statistics, convergent and discriminant validity, and reliability).

### 4.1. Construction of Items

Deductive and inductive reasoning formulated the initial seventeen RFB and nine ERD lists of scale items (Hinkin, 2005; Hinton & Platt, 2018; Morgado et al., 2017) (see Appendix A). A comprehensive literature review informed the creation of the initial items. This included reviewing the literature on social relationship satisfaction (Fincham & Beach, 2006; Fuller-Iglesias, 2015; Mellor et al., 2008; Sabatelli, 1988), intimate relationship success (Apostolou, 2015; Awruk et al., 2026; Fletcher et al., 1999; Overall & Hammond, 2018), happy couples and singles (B. DePaulo, 2017; B. M. DePaulo & Morris, 2005; Oh et al., 2022; Walsh et al., 2023), single self-love wellbeing (Harshad & Ghosh, 2022; Henschke & Sedlmeier, 2023; Jansen, 2017), expectations in social relationships (Akhter-Khan et al., 2023; Ladbury & Hinsz, 2018; Lemay & Venaglia, 2016; Millar & Tesser, 1988), social relationship dissatisfaction (Røsand et al., 2014; Velthorst et al., 2024; Whisman et al., 2000), and close relationship disappointment letdown (Kayser & Rao, 2013; Miller-Day & Lee, 2001; Peel & Caltabiano, 2021). Marketing experts and social psychologists discussed items for content relevancy and quality. This aided cross-referencing terms and phrasing.

The literature included psychiatric and family therapy studies that closely investigated individuals in relationships. Intimate relationships with others and within oneself are a basic human need well studied in nonbusiness contexts. Unmet expectations and dissatisfaction in social relationships translate into gaps in a sense of connectedness (Alabri, 2022; Brown & Kuss, 2020). When there are these gaps, individuals seek ways or products to fill this deficit (e.g., buying an influencer’s recommended product to feel like a part of a community) (Beyens et al., 2016; Dinh & Lee, 2022; N. M. Lambert et al., 2013). This project extends understanding of relational needs to construct generalizable psychometric measures that can apply to those who are asexual, single, seeking relationships, in relationships, and so forth. Humans have a need for love and connection, even if it is one deep relationship with a platonic friend. These measures open opportunities for researchers to investigate relational beliefs on consumer behaviors.

### 4.2. Data Collection and Participants

The high-quality data collection platform (CloudResearch) was utilized to collect a large dataset (Douglas et al., 2023) (see Table 1). The convenience sample represented the diverse U.S. general population (Coppock & McClellan, 2019; Levay et al., 2016). Compared to other platforms, CloudResearch participants showed proficient attention, reliability, honesty, and comprehension (Peer et al., 2022). Online surveys allow the ability to add requirements to complete all survey section questions (Ebert et al., 2018). Diverse and large representative samples reduce potential confounds from online surveying (Ball, 2019; Terhanian et al., 2016). Urban, suburban, and rural participation was represented across the U.S. based on self-reported ZIP codes. The survey started with online consent followed by demographic questions and measures. The survey included attention check questions to help assess for data quality (Buhrmester et al., 2016; Ward et al., 2017). Nine hundred and twenty-three participants initially completed the survey. Fourteen participants did not complete the survey. Attention checks were not passed by eleven participants (e.g., selecting a specific choice within a survey section). Analysis was performed on those remaining (*N* = 898). About half of the participants identified as male (*n* = 430) and female (*n* = 468). Two hundred and sixty-seven participants identified as single. Approximately seven hours was the median number of hours of social media used each week.

### 4.3. Inter-Item Correlations and Reduction

Inter-item correlations indicated that eight of seventeen initial items (from the RFB items) and three of nine initial items (from the ERD items) did not demonstrate adequate values (|r|s < 0.30) (Tabachnick et al., 2007). The researchers removed the eight and three respective items below the threshold from the initial list of items (Worthington & Whittaker, 2006). For example, “Deep connection with someone fills emotional gaps that someone cannot handle alone” showed insufficient correlation with the other RFB items. A reduction in the item list was based on the statistical results (Boateng et al., 2018). Reducing a diverse and large set of initial construct items decreases odds of missing suitable construct items (Flight et al., 2011; Pommer et al., 2013). Kaiser–Meyer–Olkin’s measure of sampling adequacy and Bartlett’s test of sphericity [RFB 0.800, *χ*^2^(15) = 2300.34, *p* < 0.001; ERD 0.671, *χ*^2^(3) = 704.49, *p* < 0.001] showed acceptable factor analysis for the refined list of items (Cerny & Kaiser, 1977; Kaiser, 1981; Tobias & Carlson, 1969).

### 4.4. Factor Loadings and Descriptive Statistics

Recommended factor loading scores were between 0.40 and 0.70, with higher scores indicating better results (Hulland, 1999; Peterson, 2000). The criteria for selecting six (of the nine remaining RFB items) and three (of the six remaining ERD items) exceeded 0.70 loading scores for the refined list of items (Chyung et al., 2017; Hair et al., 2010, p. 125) (see Table 2). For the RFB items, scree plot and parallel analysis showed appropriateness with two factors. The first factor explained 53.8% of the total variance, while the second factor explained 21.4% of the total variance. The two factors collectively captured 75.2% of the total variability in the dataset. Direct oblimin rotated analysis with two principal factors generated factor loading scores above 0.20 for the second factor (Abdi, 2003). For the ERD items, a scree plot and parallel analysis showed appropriateness for one factor. The single factor explained 67.7% of the total variance. Combining the list of RFB and ERD items to force them into one factor and three factors (single construct) exhibited unsatisfactory scores (Dunn et al., 1994; R. D. Gibbons et al., 1985). Two factors and six items suited the refined RFB measure. One factor and three items suited the refined ERD measure.

### 4.5. Confirmatory Factor Analysis

A CFA model assessed the measurement structure and initial construct validity using SPSS AMOS V25, including the latent variables: personal wellbeing (Andreassen et al., 2015), social comparison (F. X. Gibbons & Buunk, 1999), conscientiousness, and agreeableness (Gosling et al., 2003). The factor structure demonstrated acceptable model fit, *χ*^2^/*df* = 4.012, *p* < 0.0001, RMSEA = 0.058 (<0.07), SRMR = 0.060 (<0.08), and CFI = 0.921 (Hooper et al., 2008; Kenny, 2015; Steiger, 2007). All retained items exceeded the recommended minimum 0.40 loading threshold, ranging from 0.714 to 0.879 for RFB and from 0.595 to 0.830 for ERD (Watkins, 2018).

### 4.6. Convergent Validity, Discriminant Validity, and Reliability

Level of internal consistency was evaluated with composite reliability (CR) scores (Hair et al., 2010). Evaluating whether items measured the same underlying intended construct was assessed utilizing average variance extracted (AVE) scores (Fornell & Larcker, 1981). The CR/AVE scores for RFB factor 1 were 0.901/0.652, and for RFB factor 2, they were 0.842/0.590. The CR/AVE scores for the ERD measure were 0.835/0.534. CR scores above 0.70 are considered acceptable, and AVE scores greater than 0.50 are considered good when evaluating convergent validity (Fornell & Larcker, 1981; Hair et al., 2010). The unrealistic expectations construct demonstrated adequate composite reliability (CR = 0.817), although the average variance extracted (AVE = 0.439) was below the recommended threshold of 0.50, suggesting limited convergent validity. Despite a lower AVE value, the construct was retained because of its theoretical relevance to the conceptual model and acceptable composite reliability (Sarstedt et al., 2022).

Correlating the RFB and ERD scales with validated measures is a method to assess uniqueness (Maloney et al., 2012; Mathieu & Farr, 1991). Positive correlations display a level of convergent validity. Negative correlations display a level of discriminant validity. Correlation scores of one would show measures being not unique and no different. Correlation scores quantify the level of relatedness and differentiation between measures (Lucas et al., 1996). The RFB scale positively correlated with the ensuing scales: personal wellbeing (i.e., general wellness in health, relationships, and achievements) (*r* = 0.209, *p* < 0.001) (e.g., “Overall in life, how satisfied are you with each of the following?… Your personal relationships?”) (alpha = 0.80) (Andreassen et al., 2015), unrealistic expectations (i.e., high expectations in others to cater to one’s wellbeing) (*r* = 0.386, *p* < 0.001) (e.g., “I always want people to show understanding to me”) (alpha = 0.66) (Hamamci & Büyüköztürk, 2004), social comparison (i.e., evaluating one’s abilities and opinions compared to others) (*r* = 0.146, *p* < 0.001) [e.g., “I often compare how my loved ones (boy or girlfriend, family members, etc.) are doing with how others are doing”] (alpha = 0.83) (F. X. Gibbons & Buunk, 1999), conscientiousness (i.e., organized and responsible) (*r* = 0.081, *p* < 0.05) (e.g., “Dependable, self-disciplined”) (alpha = 0.50) (Gosling et al., 2003), and self-esteem (i.e., one’s sense of self-worth) (*r* = 0.129, *p* < 0.001) (e.g., “I have high self-esteem”) (test–retest reliability = 0.64) (Robins et al., 2001).

The RFB scale negatively correlated with the ensuing scales: agreeableness (i.e., trusting and cooperative) (*r* = −0.042, *p* = 0.210) (e.g., “Sympathetic, warm”) (alpha = 0.40) (Gosling et al., 2003) and amount of stress (i.e., perceived level of personal strain in the past year) (*r* = −0.142, *p* < 0.001) (e.g., “In the past year, how would you rate the amount of stress in your life (at home and at work)?”) (test–retest reliability = 0.66) (Littman et al., 2006).

Meanwhile, the ERD scale positively correlated with the ensuing scales: social comparison (*r* = 0.011, *p* = 0.744) and amount of stress (*r* = 0.229, *p* < 0.001). The ERD scale negatively correlated with the ensuing scales: personal wellbeing (*r* = −0.197, *p* < 0.001), unrealistic expectations (*r* = −0.203, *p* < 0.001), conscientiousness (*r* = −0.145, *p* < 0.001), self-esteem (*r* = −0.130, *p* < 0.001), and agreeableness (*r* = −0.024, *p* = 0.481).

Good internal reliability was demonstrated by the scales in the development sample [six-item RFB (alpha = 0.820), three-item ERD (alpha = 0.757), and four-item current relational satisfaction (alpha = 0.806)]. Precision among the items for each construct was observed based on these reliability scores (Avalos et al., 2005; Kwon et al., 2013; Robbins et al., 2010).

### 4.7. Test–Retest Reliability

Test–retest reliability was conducted using averages of the construct items (see Table 3). Reliability was assessed using a two-way, mixed-effects, single-measure intraclass correlation coefficient [ICC(3,1)], with the consistency option selected (Cicchetti, 1994; Koo & Li, 2016). This method assessed relative stability of responses between time one and two (i.e., six to eight days later). This approach bypassed systematic shifts in mean scores. The six-item RFB scale (ICC = 0.650, 95% CI: 0.507 to 0.758) exhibited adequate consistency at the 95% confidence interval. The ERD scale (ICC = 0.670, 95% CI: 0.534 to 0.773) exhibited adequate consistency at the 95% confidence interval. Pooled standard deviation ranged between 0.975 and 1.166. Standard error of measurement ranged from 0.576 to 0.668, displaying reasonable measurement precision. Overall, the measures displayed adequate relative stability over time.

### 4.8. Common Method Bias

Procedural steps were taken to minimize common method bias at the survey design stage, including random presentation of items within each scale section, participant anonymity, and confidentiality of responses as established in the consent process (Memon et al., 2023). Common method bias was assessed using the development sample, given this sample contained all focal construct items. Harman’s single-factor test was conducted by loading all final retained scale items from all four focal constructs into a single principal component factor analysis with no rotation (Podsakoff et al., 2003). The largest emerging factor accounted for 34.5% of the total variance (below the 50% threshold), indicating common method bias was not a substantial concern with the sample.

## 5. Stage 2: Independent CFA Validation

### 5.1. Participants and Data Collection

Stage 2 utilized a convenience sample recruited through CloudResearch independent from stage 1’s development sample (*N* = 223). Stage 2 aimed to replicate and cross-validate the measurement structure identified in the stage 1 development sample.

### 5.2. Confirmatory Factor Analysis

CFA was conducted in SPSS AMOS V25 on the independent validation sample to confirm the factor structure identified in stage 1 (Arbuckle, 2014; Jackson et al., 2009). The same latent variable CFA model from stage 1 was tested in an independent validation sample to examine consistency of the measurement structure and construct validity. The factor structure demonstrated acceptable model fit, *χ*^2^/*df* = 1.947, *p* < 0.0001, RMSEA = 0.065 (<0.07), SRMR = 0.075 (<0.08), and CFI = 0.892 (Hooper et al., 2008; Kenny, 2015; Steiger, 2007). All retained items exceeded the recommended minimum 0.40 loading threshold, ranging from 0.720 to 0.834 for RFB and from 0.575 to 0.790 for ERD (Watkins, 2018) (see Table 4).

### 5.3. Convergent Validity, Discriminant Validity, and Reliability

Convergent validity was supported in the independent validation sample by satisfactory composite reliability and average variance extracted values (Fornell & Larcker, 1981; Hair et al., 2010). The CR/AVE scores for RFB factor 1 were 0.887/0.684, and for RFB factor 2, they were 0.819/0.512. The CR/AVE scores for the ERD measure were 0.828/0.501.

Discriminant validity was assessed using the Fornell–Larcker criterion and HTMT ratios (Henseler et al., 2015) (see Appendix B). In both the development and validation samples, the square root of average variance extracted (AVE) for each construct exceeded its corresponding inter-construct correlations, supporting discriminant validity. For example, in the development sample, the value for RFB factor 1 (0.808) and RFB factor 2 (0.768) exceeded their inter-construct correlation (*r* = 0.346). Similar patterns were observed in the validation sample, with adequate separation between constructs (e.g., RFB factor 1 = 0.827; RFB factor 2 = 0.716; *r* = 0.362). HTMT ratios were below the recommended threshold of 0.85 across all construct pairs in both samples, ranging from 0.390 to 0.660 in the development sample and 0.360 to 0.600 in the validation sample, providing additional evidence of discriminant validity.

Cronbach’s alpha values were acceptable for RFB (alpha = 0.801) and ERD (alpha = 0.708).

### 5.4. Comparison with Stage 1 Development Sample

The CFA results in the independent validation sample showed consistency with the stage 1 development sample psychometric results across key indicators. Fit indices, AVE, CR, and alpha values demonstrated consistency within acceptable ranges across both samples. Minor differences in fit indices between samples may be attributed to sample size differences and participant composition (Vandenberg & Lance, 2000). Taken together, these comparative findings provide independent psychometric support for the RFB and ERD scales between two separate samples. Composite reliability and AVE values showed consistency across stage 1 and stage 2, indicating stability of the measurement model across independent samples (see Table 5).

## 6. Stage 3: Predictive Validity Serial Mediation Analysis

Predictive validity analysis utilized the original development sample.

### 6.1. Measures

Independent variable. Rescue fantasy beliefs (RFB) measures individuals’ belief that a close social relationship will deliver a sense of “happily ever after” by resolving loneliness, life struggles, and deficits in self-worth on two factors using six items (refined in the previous psychometric development sections) [e.g., (1) support expectancy “A charming prince or princess in someone’s life will save them from life’s challenges” and (2) self-worth enhancement “A close relationship positively lifts how someone views themselves”] (see Appendix C). This is measured on a seven-point scale (1—strongly disagree to 7—strongly agree).

Mediator variables. Expected relational disappointment (ERD) measures individuals’ belief that social relationships will fail to resolve life challenges, loneliness, and distress using three items (refined in the previous psychometric development sections) (e.g., “Even in a close relationship, it can be as lonely as when someone is single”) on a seven-point scale (1—strongly disagree to 7—strongly agree).

Current relational satisfaction measures individuals’ sense of contentment with their current close social relationships in four aspects [“Rate how dissatisfied/satisfied you are with your close relationship(s) on the following qualities” (1) positivity, (2) safety, (3) adventure, (4) trust]. This is measured on a seven-point scale (1—very dissatisfied to 7—very satisfied). Internal reliability was high in the current study (alpha = 0.806). The measure used the term “close relationship(s)” to encompass a wider range of individual differences in social needs (e.g., persons who are single, married, and asexual). Current relational satisfaction was operationalized with transparent and direct items capturing the target construct, which is consistent with established psychometric criteria for brief measures (P. Kline, 2015; Nunnally & Bernstein, 1994; Rossiter, 2002). The measure reflects attachment theory’s conceptualization of relational satisfaction as a generalized appraisal of close relationship quality rather than an evaluation of a specific romantic partnership (Bowlby, 1980; Mikulincer & Shaver, 2010). Research demonstrated that attachment-related relational schemas operate across three relationship domains: family, platonic friendships, and romantic partners (Overall et al., 2003). This operationalization is intentionally inclusive and consistent with attachment theory (Thompson et al., 2022).

Dependent variable. Shopping addiction measures individuals’ preoccupation with shopping/buying behaviors and perceived adverse effects on their wellbeing using seven items (e.g., “I shop/buy so much that is has impaired my well-being”) on a five-point scale (1—completely disagree to 5—completely agree) (alpha = 0.87) (Andreassen et al., 2015).

Control variables. Path analysis included gender, relationship status (single), hours on social media (weekly average), and BMI (body mass index) as control variables. There was dummy coding of gender (male = 1, female = 2). Among clinical samples, females were associated with greater compulsive buying compared to males (Nicoli de Mattos et al., 2016; Tetzlaff et al., 2026). Meta-analytic review found women reported higher compulsive buying rates than men across diverse samples (Maraz et al., 2016). Single relationship status associated with greater romantic and family loneliness (from lower perceived social support from significant others) compared to partnered individuals (Adamczyk, 2016). Loneliness directly associated with shopping addiction, mediated by depression (Rachubińska et al., 2023). Social isolation and unmet connection needs are associated with compulsive online buying behaviors (Wang et al., 2021). Social media addiction associated with higher shopping addiction behaviors compared to individuals classified as low risk for social media addiction (Tullett-Prado et al., 2023). Social media use is associated with compulsive shopping behavior, mediated by materialism (Jameel et al., 2024). Social commerce environments with reinforcing rewards can increase emotionally driven purchases that support compulsive buying habits (Dang et al., 2025). BMI (body mass index) was included as a control variable based on prior empirical and theoretical support showing BMI associating with compulsive buying behavior (Sansone et al., 2013). Social pressures relating to body-related image may represent a potential source of variance with shopping addiction (Azevedo & Azevedo, 2023). Negative perceptions of one’s physical appearance were shown to produce negative emotions and activate self-regulatory compensatory behaviors, such as impulsive buying (Cai et al., 2021).

### 6.2. Predictive Validity Results

**Figure 2:**
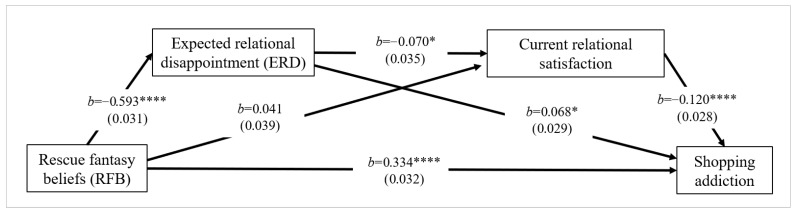
Serial mediation modeled effects on shopping addiction. Notes: Control variables included gender, single (relationship status), hours on social media (weekly average), and BMI (body mass index). * = *p* < 0.05, ** = *p* < 0.01, *** = *p* < 0.001, **** = *p* < 0.0001. Additional asterisks and corresponding *p*-values were included in these notes for consistency across tables and figures.

Descriptive statistics and correlations are illustrated in Table 6. Moderate normality thresholds were observed for variables with kurtosis values within ± 7.0 and skewness values within ± 2.0 (R. B. Kline, 2015; Wong, 2016). Serial mediation analysis was performed using SPSS PROCESS V3.5 (model 6) with mean centering and the 10,000 bootstrapped sampling procedure with the stage 1 development sample (Hayes, 2012, 2017; Preacher et al., 2007) (see Table 7 and Figure 2). SPSS AMOS V25 serial mediation model analysis of fit indexes demonstrated good fit for the large sample size, *χ*^2^/*df* = 3.986, *p* < 0.0001, RMSEA = 0.058 (<0.07), SRMR = 0.026 (<0.08), and CFI = 0.968 (Hooper et al., 2008; Kenny, 2015; Steiger, 2007). RFB was related to higher shopping addiction (*b* = 0.334, *t* = 10.430, *SE* = 0.032, *p* < 0.0001 [*LLCI* 0.2713 *ULCI* 0.3971]) (support for H1).

RFB was associated with lower ERD (*b* = −0.593, *t* = −18.910, *SE* = 0.031, *p* < 0.0001 [*LLCI* −0.6548 *ULCI* −0.5317]) (support for H2). ERD was associated with higher shopping addiction (*b* = 0.068, *t* = 2.352, *SE* = 0.029, *p* < 0.05 [*LLCI* 0.0113 *ULCI* 0.1249]) (support for H3). Bootstrap analyses (10,000 samples) showed a significant indirect effect of RFB on shopping addiction through ERD (*b* = −0.040, *SE* = 0.020 [*LLCI* −0.0804 *ULCI* −0.0002]). RFB did not significantly associate with lower current relational satisfaction (*b* = 0.041, *t* = 1.056, *SE* = 0.039, *p =* 0.292 [*LLCI* −0.0352 *ULCI* 0.1170]) (lack of support for H4). Current relational satisfaction associated with lower shopping addiction (*b* = −0.120, *t* = −4.336, *SE* = 0.028, *p* < 0.0001 [*LLCI* −0.1744 *ULCI* −0.0657]) (support for H5). Bootstrap analyses (10,000 samples) showed a nonsignificant indirect effect of RFB on shopping addiction through current relational satisfaction (*b* = −0.005, *SE* = 0.005 [*LLCI* −0.0155 *ULCI* 0.0046]).

ERD was associated with lower current relational satisfaction (*b* = −0.070, *t* = −2.000, *SE* = 0.035, *p* < 0.05 [*LLCI* −0.1385 *ULCI* −0.0013]). Bootstrap analyses (10,000 samples) showed a significant serial indirect effect of RFB on shopping addiction through ERD and current relational satisfaction (*b* = −0.005, *SE* = 0.003 [*LLCI* −0.0108 *ULCI* −0.0002]) (support for H6). The total effect of RFB on shopping addiction was significant (*b* = 0.284, *t* = 10.357, *SE* = 0.027, *p* < 0.0001 [*LLCI* 0.2301 *ULCI* 0.3377]), showing higher RFB associated with higher shopping addiction. When accounting for both mediators, the direct effect remained significant (*b* = 0.334, *t* = 10.430, *SE* = 0.032, *p* < 0.0001 [*LLCI* 0.2713 *ULCI* 0.3971]), indicating partial mediation.

Control variables demonstrated nonsignificant results, except in three instances in the model. Females (compared to males) are associated with higher ERD (*b* = 0.212, *t* = 3.148, *SE* = 0.067, *p* < 0.01 [*LLCI* 0.0799 *ULCI* 0.3446]) and higher shopping addition (*b* = 0.238, *t* = 4.068, *SE* = 0.059, *p* < 0.001 [*LLCI* 0.1232 *ULCI* 0.3529]). Single relationship status (compared to those in other relationship statuses) is associated with lower current relational satisfaction (*b* = −0.238, *t* = −3.122, *SE* = 0.076, *p* < 0.01 [*LLCI* −0.3868 *ULCI* −0.0882]). Meanwhile, hours on social media and BMI did not associate with any outcomes in the model.

## 7. Discussion

### 7.1. General Discussion

The results extend the ability to study attachment theory and how consumers seek to fulfill relational needs with consumer goods. Attachment-related relational schemas guide how individuals seek security and comfort in attempts to fulfill social needs (Bowlby, 2008). Serial mediation main effects showed that RFB was positively associated with shopping addiction (H1). This indicates how illusionary social beliefs can spur buying behaviors to support a better-than-reality internal narrative. For example, products often offer symbolic value such as brand association, which increases one’s self-worth (Barnes, 2003; Cho et al., 2015). This supports prior research that found insecurely attached adults to be more likely to use indirect or symbolic coping strategies versus direct relational repair (Mikulincer & Shaver, 2010). Researchers found individuals develop emotional attachments to brands even though brands are often curated social constructs (Thomson et al., 2005). For instance, wearing a branded clothing line can translate into membership with a fitness community (e.g., Lululemon) (Y. Guo et al., 2024; Qian & Zou, 2021). The RFB scale provides a useful tool to capture a unique cognitive–motivational strategy used to manage unmet attachment needs (Kobak et al., 2016). In contrast to traditional attachment measures, the RFB scale measures how individuals envision future relationships rather than those of the past (e.g., measures identifying persons with an insecure attachment style) (Overall & Simpson, 2015). RFB attributes savior qualities to others to bring lasting happiness, companionship, and security. RFB are a relational form of the arrival fallacy (e.g., “If only I had a best friend, then I will not be lonely”). This advances attachment research with a tool to study illusionary coping fantasies that influence downstream consumption patterns. This contributes to how marketing researchers can investigate how consumers gain perceived psychological benefits through buying.

Furthermore, the findings reiterate that shopping addiction is considered a maladaptive emotional regulation behavior (Dittmar, 2005b). Insecure attachment was linked to greater reliance on external regulators to cope with high levels of stress (Mikulincer et al., 2003). The positive relationship between RFB and shopping addiction suggests how these consumers are more vulnerable to partake in maladaptive buying behaviors. For example, RFB from lacking self-regulatory control can contribute to thoughtless spending (e.g., compulsive buying) (Akın, 2025). This is because such behaviors are sometimes driven by emotional dysregulation and a lack of control as a coping strategy (Ridgway et al., 2008). For example, shopping is a behavior that individuals can exhibit control and feel positive doing, even though it does not address unmet relational needs (Black, 2006). This is like the illusionary belief that if someone buys a new outfit, others will take notice and care to become closer friends (Cho et al., 2015). This extends previous research by constructing an attachment-related measure that was found to increase shopping addiction (rather than a broader developmental insecure attachment style). This highlights how emotional dysregulation can boost responsiveness to symbolic and aspirational marketing (e.g., an image of a happy couple wearing an expensive ring). Products can act as an extension of oneself and fill gaps in one’s self-concept (R. Belk, 2016; R. W. Belk, 2013). Products can help someone feel better about their current situation by providing psychological benefits to support internal beliefs (e.g., feel taller and more confident with shoe lifts) (Taylor et al., 2000; Zimmerman, 2009).

Additionally, the results showed that higher RFB was associated with lower ERD, which aligns with attachment theory (H2). Attachment theorists described how relational distress is internally regulated when directly attempting to change relationships is uncertain or risky (Ainsworth et al., 2015). For example, when a parent is consistently dismissive of a child’s requests (e.g., getting a piercing or certain attire to fit in with friends), the child may turn inward to self-soothe through healthy or unhealthy coping methods. This also establishes opinions of the relationship as unsupportive to meet one’s needs. Someone may believe “Why even try, if they constantly say no?” Concerns of an argument or disrupting an already tenuous relationship are not worth the effort. This supports prior research that found perceived violations in relational expectations stimulate emotional disappointment (Hendrick, 1988; Miller-Day & Lee, 2001). This differs from pure objective relationship quality. It is evaluations of relational expectations and outcomes that also foster impressions of the relationship quality (Canevello & Crocker, 2010). RFB can function as an anticipatory regulation strategy to preserve hope and suspend negative evaluations of current relationships (Carver & Scheier, 2014). Carver and Scheier (2014) explain how relationship optimism increases sustained effort to navigate challenges and invest in social relationships. For instance, if someone believes a close relationship will save them in difficult times, they are more likely to place positive attributes and effort on the relationship. This implies that future-oriented beliefs (i.e., RFB) can reduce relational disappointment with psychological reinterpretations (rather than reaching interpersonal resolutions) (Shaver & Mikulincer, 2002). This is like believing that a movie was better than it really was because of a favorite actor in the movie. While fragile in supporting long-term relational wellbeing, psychological expectations play a role in shaping emotional evaluations of relationships. Notably, lower disappointment may reflect emotional disengagement (Nichols et al., 2015). This can appear as withdrawal and settling for a subpar relationship as it is, while appreciating the few positive aspects that fill certain needs. For instance, a partner may be disappointed for not performing household chores, but this is tolerated for their financial household support from holding a stable job. This maintains someone’s RFB in others, while downplaying negative aspects of the relationship. This upholds one’s relational arrival fallacy cognitive narrative. In other words, a savior is improving my wellbeing and happiness because that is what I expect them to do. This aligns with attachment theory research that found insecure attachment styles to maladaptively regulate affect and behave in intimate relationships (particularly under stress) (Simpson & Rholes, 2017). RFB appears to help regulate feelings about their relationships, despite leaving negative aspects of a core relational structure unchanged.

### 7.2. Theoretical Implications

Serial mediation analysis supports and extends attachment theory on compensatory consumer behavior. The results showed that ERD was associated with higher shopping addiction (H3). Additionally, current relationship satisfaction was negatively associated with shopping addiction (H5). This builds on prior qualitative research that found self-image, identity construction, and emotional regulation are closely tied to excessive consumption (Dittmar & Drury, 2000). For instance, when consumers buy fashion products (e.g., clothing or handbags), they offer emotional comfort and symbolic meaning that supports one’s self-identity (Hauck & Stanforth, 2007). They do not simply provide utilitarian value. Many individuals own more clothes that surpass their utilitarian needs in the modern industrial world. Unmet relational needs can motivate emotions, behaviors, and relationships in attempts to fulfill them (Baumeister & Leary, 1995). Secure relationships can lower reliance on material substitutes for emotional fulfillment (Pieters, 2013). Individuals seek emotional attachment bonds that are reliable, responsive, and secure (Bowlby, 1969; Hazan & Shaver, 2017). Relational disappointment acts as an emotional link between attachment and consumption (Rucker & Galinsky, 2016). Rucker and Galinsky (2016) imply that when relational disappointment is present, it activates a relationship-oriented consumption mindset. Consumers may seek avenues like brand association or buying behaviors to cope. We contend that buying acts as a self-soothing strategy when there is a sense of insecurity and disconnect from attachment figures. This suggests that relationship quality is a meaningful risk factor for addictive buying behavior. Buying behaviors can serve deeper psychological benefits to compensate for dissatisfaction and disappointment in relational attachments.

The key findings showed that RFB on shopping addiction was mediated through ERD and current relational satisfaction (H6). Expectations in attachment figures (e.g., close relationships) can be met or unmet (Vannier & O’Sullivan, 2018). When there is a gap in unmet expectations and relationship quality declines, individuals tend to turn towards methods to cope with unmet relational needs (e.g., excessive shopping) (Vangelisti et al., 2002). This illusionary RFB in others sets up a common predicament of disappointment because many people have an innate self-interest motive (Miller, 1999). Self-interest is a survival instinct (e.g., on a plane, put on one’s own oxygen mask before helping another) (Maitland, 2002). Savior expectations in strangers to alleviate one’s loneliness and solve life’s challenges tend to be wishful thinking. Expecting a charming prince or princess to arrive is often what the assumed charming person is also imagining. For example, on the first date, people often envision higher prospects for others than what is real. People expect others to bring happiness into their lives, when long-term happiness is frequently built through joint communal effort (Abele, 2014; Lu & Argyle, 1991). Time and effort help to build long-term close relationships (Eastwick et al., 2018). After which, working together can solve common problems and reduce feelings of loneliness (not necessarily the random hero that arrives in one’s life in shining armor) (De Jong Gierveld & Fokkema, 2015). There tend to also be ruptures and disagreements in relationships that separate close ties (Fingerman et al., 2004). People disappoint by misunderstanding and having different values (Mortensen, 2006). For example, some people highly value flowers as gifts, while others may view them as a waste of money. Some people expect friends to share deep secrets together, while others are happy with superficial relationships. Some people want to do everything together, while others desire space and independence. Matching values and shared interests are some basic building blocks to happy relationships (Gottman & DeClaire, 2017; Sagawa & Segal, 2000). The findings support how developmental social attachments form relational schemas and beliefs (i.e., RFB). This implies that savior expectations in others can be misattributed to others as an aspirational coping strategy to underlying unmet relational needs. This sets up high expectations in others, that when frequently unmet, can elicit alternative coping outlets (e.g., shopping addiction). Thereby, RFB (i.e., a relational form of the arrival fallacy) partially outsources wellbeing to others, hoping it will replace internal unmet needs. This exposes oneself to the negative realities of relationships, without internally developing healthy self-regulatory skills.

Furthermore, this opens new avenues of attachment theory research. Attachment theorists explain that relational schemas influence expectations of availability and responsiveness in others as adults (Feeney, 2002). When expectations are repeatedly not met, feelings of disappointment can reinforce negative relational beliefs (Arriaga et al., 2018). In fact, Arriaga et al. (2018) explained how behaviors from those with an anxious attachment style seeking greater interdependence can set up disappointment from others. For example, clinging behaviors that suggest a lack of relational commitment (e.g., need for 24/7 location sharing or communication every waking hour) can cause the close tie to pull away. Even though the insecurely attached individual is seeking reassurance, such behaviors can be interpreted as a lack of trust and taking away someone’s independence (Skeen, 2014). This can maintain a cycle of abandonment or friendships that remain a safe distance away to avoid becoming too close (Gillis, 2025). This project psychometrically developed two tandem scales (i.e., RFB and ERD scales) that allow researchers to study their subsequent effects on decision-making processes. Relational schemas are strong motivators because they aim to fulfill basic human needs for social connection, belongingness, security, and love (Holmes, 2000).

### 7.3. Managerial Implications

The following managerial implications are offered as directional guidance and warrant additional empirical validation to support broader application (Cohen, 2016). The direct association between RFB and shopping addiction implies consumers may use purchases as symbolic substitutes for unmet attachment-related needs (Dittmar, 2005b) (H1). From a managerial perspective, this suggests emotionally driven consumers are more likely to be influenced by lofty promises of transformation. Psychological distress and impaired self-regulation are part of the nomological network that relates to compulsive buying (Ridgway et al., 2008). Prior research found compulsive buying to relieve negative emotional episodes (Müller et al., 2012). Brands that emphasize aspirational changes may reinforce unhealthy buying behaviors among emotionally dysregulated consumers from attachment-related issues. Research found greater incongruence in one’s ideal and actual self associated with higher materialism and compulsive shopping (Dittmar, 2005a). Materialism positively mediated the relationships between identity confusion and compulsive buying (Claes et al., 2016). This suggests that individuals may try to discover or improve their self-identity in others. However, if relationships do not suffice, material goods may be purchased to fill this void. Thereby, consumers with higher RFB are likely more susceptible to products marketed with greater symbolic meaning that help bridge discrepancies in one’s identity. Some individuals justify dysfunctional partnerships to feel safer having another body present (rather than being alone with one’s emotions) (Engel, 2023, p. 107). Such a partner serves more like a security blanket than someone valued for their individuality and personal characteristics. The reason for being together is built on savior or protection benefits. This contrasts with characteristics found among happy long-term mates (e.g., personality match and shared common interests, among other factors) (Schwartz, 2006; Zentner, 2005). Businesses can offer products that help address unresolved underlying attachment-related issues. For example, artificial intelligence (AI) smartphone counseling apps provide guided support to face attachment-related feelings (Gamble, 2020). Such apps promote doing the inner psychological work to reduce attachment-related emotions that trigger coping behaviors (Yang et al., 2025). This can help individuals identify common emotions triggered by dysregulating events and develop healthy coping practices.

The results showed that RFB was negatively associated with ERD, indicating that imagining others saving someone from life’s challenges may buffer perceived interpersonal shortcomings (H2). When higher ERD exists, consumers are more likely to exhibit shopping addiction (H3). Individuals often exhibit different practices to self-regulate intimate relationship conditions (Overall et al., 2006). Focusing on the excellent qualities of a partner can reduce attention placed on a partner’s poor qualities. This modifies one’s interpretation of events to reduce experienced negative emotions (Diefendorff et al., 2008; O’Connor, 2003). This also raises certain qualities to meet someone’s expectations, while downplaying other qualities (Sugden, 2000). Sugden (2000) explains that by changing one’s expectations, the redirected focus of attention can maintain or support motivation to perform certain activities. For instance, friends that stay together despite disrespecting and ridiculing each other often reinterpret it as having fun. The alternative of being alone can be weighed disproportionately to justify unhealthy friendships. Consumers high in RFB may reinterpret relational strain with a more favorable lens (Mikulincer et al., 2003). Buying as a coping strategy helps explain why some consumers express emotional comfort from brands despite unstable personal relationships (Park et al., 2006). Buying high status goods can help regulate low self-esteem and negative self-image (Sivanathan & Pettit, 2010). The results show how RFB can temporarily stabilize relational schemas without resolving underlying attachment needs. Avoiding problems by shifting cognition into imaginary thoughts and forming attachments to material objects can reduce the need to face unmet relational needs (Cramer, 2006; Fonagy & Target, 2007). Objects provide a sense of security, in the absence of relational needs that would otherwise be provided through social support and relational presence (Rindfleisch et al., 2009). Businesses can encourage community use of products to foster genuine connections. For example, card games (e.g., Pokémon trading card game) with in-person events have connected consumers across the globe with a common interest (Assunção et al., 2017). It brings together consumers with the possibility to form meaningful connections outside of the game. This illustrates how objects that provide little utilitarian value for survival can fill human needs (e.g., relational connection and belonging). This promotes ongoing community connection, as opposed to single encounters with a supposed lasting happy ending (i.e., relational arrival fallacy). Businesses can design products/services that directly serve unmet social needs (e.g., spike ball and escape rooms), instead of creating products that act as substitutes (Janikula, 2020; O’Szabo et al., 2022; Shakeri et al., 2017).

The results showed that RFB relates to shopping addiction through a sequential chain involving ERD and current relational satisfaction (H6). This indicates consumers in this predicament are less likely to shop for tangible needs but rather to fill a relational void (Niedermoser et al., 2021). Consumers can form emotional and psychological relationships with company brands (Thomson et al., 2005). High levels of brand attachment were found to relate to impulsive and obsessive–compulsive buying (Japutra et al., 2022). Brands and companies form longer-term relationships with consumers through loyalty programs (Chou, 2013). Loyalty programs reward repeated purchases, which unethically may exploit consumers’ relational needs (Pez et al., 2017). Loyalty programs reinforce purchase behaviors through incentives (e.g., reward points or bonus miles) (Chen et al., 2021). Loyalty programs with monetary promotions can induce impulsive purchasing behaviors (Nishio & Hoshino, 2024). Hypothetically, the more relationally dissatisfied a consumer is, the more they shop and accumulate loyalty points, which reinforces compulsive shopping (L. Guo et al., 2023). This excessive shopping can harm consumer’s psychological and financial wellbeing without addressing underlying relational needs (Davenport et al., 2012). Brands committed to ethical practice and the long-term wellbeing of their customers can tie rewards programs with socialization. For example, airline loyalty programs offer access to airport lounges (Han et al., 2012). Messages can cue social interaction with fellow travelers such as welcome signs (e.g., “Smile, you are a world traveler. Share a happy memory with a fellow explorer.”) or a turn-based in-app game with conversation starters to spur thoughtful discussions among partners. This helps promote healthy conversations that can be awkward to start, but individuals may want to have in leisure environments (Brajša-Žganec et al., 2011). This can also improve brand loyalty because emotional connections formed with others become part of the affiliation (e.g., memory of meeting a new friend at the airport lounge). Strategically prompting socialization can be low-cost and help to serve underlying long-term needs of RFB consumers.

### 7.4. Limitations and Future Research

Cross-sectional studies have potential causal inference limitations as a research design that can pertain to attachment relational studies (Maxwell & Cole, 2007). Attachment theory emphasizes developmental sequencing where relational schemas form over time and affect downstream outcomes (e.g., relational expectations and placement of beliefs on interpersonal relationships) (Sroufe et al., 2005). Prior advancements in attachment theory have used longitudinal studies to study stability of attachment-related schemas (Fraley & Roisman, 2019). A longitudinal design could test the stability of RFB as a representation of attachment expectations in interpreting relational experiences. For instance, longitudinal research found insecure attachment styles (i.e., avoidant or anxious) associated with higher expectations, higher conflict, and lower satisfaction in relationships (Roisman et al., 2005). Decades of longitudinal research on marital relationship quality and stability found marital satisfaction to change over time (Karney & Bradbury, 1995). Karney and Bradbury (1995) found early stress and expectations affect long-term relationship quality. Ongoing disappointment and conflict can culminate, eroding marital satisfaction. Attachment-related expectations can alter as close relationship interactions and emotional responses occur (e.g., early honeymoon phase of marriage versus later years) (Davila et al., 1999). Longitudinal research found adult attachment orientations to remain relatively stable and can typically change gradually with experiences (Fraley et al., 2011). The studies suggest that attachment beliefs (e.g., expectations in others to rescue someone) temporally precede evaluations in others and outcomes (e.g., current relational satisfaction). The present study performed psychometric test–retest reliability analysis, though not for predictive validity analysis. A longitudinal design in future research could buttress how attachment processes affect consumer behavior outcomes, like how unhealthy shopping behaviors are used to cope with unmet social needs (Pieters, 2013).

The results should be interpreted within the bounds of empirical support. The results indicated a lack of empirical support for the hypothesized direct relationship between RFB and current relational satisfaction (H4), despite prior research suggesting that unmet relational needs can decrease relationship quality (Hazan & Shaver, 2017). The explained variance through current relational satisfaction was also modest (*R*^2^ = 0.031). This can be attributed to how relational satisfaction is a complex and multifaceted, which the set of variables only partially explains (Hair et al., 2019). The unexpected nonsignificant positive direction relates to marital research showing idealization, which can function as a protective mechanism in relationships (Murray et al., 2011). Individuals who idealize their partners may experience a degree of immunity to declining satisfaction. Researchers explain that optimism can help sustain relationships through challenges, while also altering objective evaluations of relationship quality (Johnson, 2015). Relationship illusions (e.g., unrealistic optimism) were found to be related to greater satisfaction and trust rather than dissatisfaction (Murray & Holmes, 1997). Future research can investigate the effects of relationship optimism on subjective versus objective evaluations of relationship quality. Relationship optimism could alter perceptions and cloud judgement when conditions are suboptimal. This can deepen understanding of attachment theory and help explain why couples remain together long after relationships are dissatisfactory. Relationship optimism may function as a mechanism that helps sustain relational engagement across both satisfying and dissatisfying relationships.

Furthermore, while attachment-based fantasies can reflect unmet relational needs, prior research found that idealized relational beliefs can exist without hindering relationship satisfaction (Murray et al., 1996). For example, someone can fantasize that their partner is better than how others view them, which can form positive impressions. This suggests that RFB can serve as an aspirational function rather than a maladaptive relational schema. Imagination and reframing can provide supportive emotional regulation without necessarily disrupting relationships (Gross, 2015). This may explain why RFB did not relate to lower current relationship satisfaction. This suggests that positive attributes placed on partners from this relational arrival fallacy thinking (i.e., RFB) can have some benefits. For example, if a relationship is a healthy match, viewing a partner as a heroic savior can deepen commitment in the relationship. Future research can expand understanding of RFB as a form of self-regulatory comfort or motivational aspirations in relationships. For instance, believing that others will bring happiness and companionship into one’s life serves as a motivational reason to reach out to strangers. This can deepen what mechanism RFB serves to benefit individuals in starting, fostering, and nurturing relationships.

## 8. Conclusions

The current project makes two integrated contributions to behavioral sciences. First, it developed and validated the rescue fantasy beliefs (RFB) scale. This provides researchers with a psychometrically validated instrument for measuring attachment-based cognitions about idealized and unrealistic relationships. Second, it tested the predictive validity of this scale within a serial mediation framework. RFB was associated with shopping addiction both directly and indirectly through relational deficits in expected relational disappointment and current relational satisfaction. Together, these contributions establish RFB as a theoretically grounded construct and, when unmet, it will relate to compulsive consumer behavior.

## Figures and Tables

**Table 1:** Demographic characteristics of participants.

Demographic Characteristics	Frequency	Percentage
Gender		
Male	430	47.9
Female	468	52.1
Relationship status (single)		
Single	267	29.7
Dating, married, other	631	70.3

**Table 2:** Item–factor loadings and descriptive statistics.

				Factor Loadings	
Item	Rescue Fantasy Beliefs (RFB)	M	(SD)	Factor 1	Factor 2
(1)	The right relationship will end everything wrong in someone’s life	2.76	(1.76)	0.879	
(2)	A fulfilling relationship will end the need for much else	3.73	(1.84)	0.730	
(3)	A charming prince or princess in someone’s life will save them from life’s challenges	2.98	(1.79)	0.810	
(4)	A close relationship positively lifts how someone views themselves	5.39	(1.14)		0.805
(5)	A close relationship will help someone feel better about themselves	5.29	(1.18)		0.788
(6)	Relationship attention helps someone feel valuable	5.51	(1.06)		0.714
	Expected relational disappointment (ERD)				
(1)	Deep relationships do not fix emotional discontent	4.81	(1.55)	0.830	
(2)	Even in a close relationship, it can be as lonely as when someone is single	4.86	(1.51)	0.595	
(3)	A close relationship does not erase one’s personal struggles	5.71	(1.31)	0.736	

Notes: For the RFB scale, factor 1 represents support expectancy and factor 2 represents self-worth enhancement. The ERD scale was unidimensional. Factor loadings can change after refining the larger initial set of scale items down to the refined list due to factor structure and communality shifts. M = mean. (SD) = standard deviation.

**Table 3:** Internal consistency (Cronbach’s α) and test–retest reliability for scales.

		Development Sample (*N* = 898)	Retest Sample Subset, *N* = 84				
Scale/Subscale	Items	Internal Reliability (Cronbach’s α)	Internal Reliability (Cronbach’s α)	Test–Retest Reliability (r)	ICC	SD (Pooled)	SEM
Rescue fantasy beliefs (RFB)	6	0.820	0.820	0.651	0.650	0.975	0.576
Expected relational disappointment (ERD)	3	0.757	0.808	0.672	0.670	1.166	0.668

Notes: Stage 1 development sample reflects internal consistency during initial psychometric development of the refined item list for each construct. The retest sample included a subset of stage 1 development sample participants who responded about six to eight days later. Higher scores indicate stronger internal reliability (α ≥ 0.70) and acceptable test–retest reliability (r ≥ 0.50) (Cicchetti, 1994; Koo & Li, 2016).

**Table 4:** Confirmatory factor analysis results (development sample).

Construct	Item	Standard Loading	CR	AVE
RFB factor 1	Item 1	0.834	0.849	0.652
	Item 2	0.781		
	Item 3	0.817		
RFB factor 2	Item 1	0.776	0.811	0.589
	Item 2	0.819		
	Item 3	0.720		
ERD	Item 1	0.787	0.774	0.534
	Item 2	0.575		
	Item 3	0.790		
Personal wellbeing	Item 7	0.820	0.902	0.648
	Item 6	0.717		
	Item 5	0.871		
	Item 4	0.730		
	Item 3	0.663		
	Item 2	0.743		
	Item 1	0.847		
Unrealistic expectations	Item 1	0.507	0.817	0.439
	Item 2	0.690		
	Item 3	0.362		
	Item 4	0.595		
	Item 5	0.651		
	Item 6	0.459		
	Item 7	0.496		
	Item 8	0.605		

**Table 5:** Composite reliability (CR) and average variance extracted (AVE) values for stage 1 and stage 2.

Construct	Stage 1 CR	Stage 1 AVE	Stage 2 CR	Stage 2 AVE
RFB factor 1	0.901	0.652	0.887	0.684
RFB factor 2	0.842	0.590	0.819	0.512
ERD	0.835	0.534	0.828	0.501
Personal wellbeing	0.912	0.648	0.903	0.567
Unrealistic expectations	0.817	0.439	0.801	0.421

Notes: CR = composite reliability. AVE = average variance extracted. Stage 1 represents the development sample. Stage 2 represents the validation sample.

**Table 6:** Descriptive statistics and cross-level correlations.

Variables		*M*	*SD*	*Skewness*	*Kurtosis*	1	2	3	4	5	6	7	8
Constructs													
1.	Rescue fantasy beliefs (RFB)	4.28	1.09	0.12	−0.26	—							
2.	Expected relational disappointment (ERD)	5.13	1.20	−0.76	0.35	−0.556 **	—						
3.	Current relational satisfaction	5.61	1.05	−1.12	1.37	0.109 **	−0.121 **	—					
4.	Shopping addiction	2.14	0.92	0.72	−0.23	0.309 **	−0.090 **	−0.112 **	—				
Control variables													
5.	Gender (female)	1.52	0.50	−0.09	−2.00	−0.168 **	0.178 **	−0.059	0.088 **	—			
6.	Relationship status (single)	0.30	0.46	0.89	−1.21	−0.082 *	0.056	−0.110 **	−0.016	−0.030	—		
8.	Hours on social media (weekly average)	7.34	3.32	−0.40	−1.13	0.099 **	−0.049	0.041	0.063	0.020	−0.003	—	
9.	BMI (body mass index)	27.20	7.45	2.23	11.41	−0.082 *	0.079 *	−0.041	0.013	0.008	−0.015	0.071 *	—

Notes: *N* = 898, * *p* < 0.05, ** *p* < 0.01 level (two-tailed).

**Table 7:** Serial mediation model predicting shopping addiction.

**Predictor**	***Coeff.***	***SE***	***t***	***p***
Expected relational disappointment (ERD) (M1)				
Rescue fantasy beliefs (RFB)	−0.593	0.031	−18.910	<0.0001
Gender (female)	0.212	0.067	3.148	<0.01
Single (relationship status)	0.041	0.073	0.558	0.577
Hours on social media	0.000	0.010	0.025	0.980
BMI	0.006	0.005	1.257	0.209
*R*^2^ = 0.318, *F*(5, 892) = 83.337, *p* < 0.0001				
Current relational satisfaction (M2)				
Rescue fantasy beliefs	0.041	0.039	1.056	0.292
Expected relational disappointment	−0.070	0.035	−2.000	<0.05
Gender (female)	−0.086	0.071	−1.211	0.226
Relationship status (single)	−0.238	0.076	−3.122	<0.01
Hours on social media	0.011	0.011	1.071	0.285
BMI	−0.005	0.005	−1.040	0.299
*R*^2^ = 0.031, *F*(6, 891) = 4.731, *p* < 0.0001				
Shopping addiction (DV)				
Rescue fantasy beliefs	0.334	0.032	10.430	<0.0001
Expected relational disappointment	0.068	0.029	2.352	<0.05
Current relational satisfaction	−0.120	0.028	−4.336	<0.0001
Gender (female)	0.238	0.059	4.068	<0.001
Relationship status (single)	0.001	0.063	0.019	0.985
Hours on social media	0.008	0.009	0.929	0.353
BMI	0.004	0.004	0.940	0.348
*R*^2^ = 0.114, *F*(7, 890) = 21.156, *p* < 0.0001				
Indirect effects (bootstrap estimates)				
**Effect**	**Coeff.**	***SE***		**95% CI**
RFB → ERD → shopping addiction	−0.040	0.020		[−0.0804, −0.0002]
RFB → current relational satisfaction → shopping addiction	−0.005	0.005		[−0.0155, 0.0046]
RFB → ERD → current relational satisfaction → shopping addiction	−0.005	0.003		[−0.0108, −0.0002]

Notes: There was dummy coding of gender, with males coded as 1 and females coded as 2. Participants indicated their current relationship status by selecting one option from a predefined list of categories. Participants who selected “Single (not in a relationship and not dating)” were dummy coded as 1, with all other options coded as 0. Hours on social media were a self-reported weekly average. BMI (body mass index) was calculated from self-reported height (feet/inches) and weight (pounds) information.

## Data Availability

Due to the nature of this research, participants in this study did not agree to have their data shared publicly, so supporting data is not available.

## Appendix A. List of Initial Rescue Fantasy Beliefs (RFB) and Expected Relational Disappointment (ERD) Scale Items

Rescue fantasy beliefs (RFB):

(1) The right relationship will end everything wrong in someone’s life.
(2) True connection with someone will resolve most emotional pain.
(3) Deep connection with someone will protect them from loneliness.
(4) A close relationship will solve inner issues one cannot fix alone.
(5) Deep connection with someone fills emotional gaps that someone can not handle alone.
(6) Loving connection will eliminate feelings of being alone.
(7) A fulfilling relationship will end the need for much else.
(8) The right relationship will end doubts of someone’s self-worth.
(9) A charming prince or princess in someone’s life will save them from life’s challenges.
(10) Closeness with another is the missing piece for emotional wellbeing.
(11) Truly being known by someone will lead to lasting happiness.
(12) A close relationship positively lifts how someone views themselves.
(13) A close relationship will help someone feel better about themselves.
(14) Relationship attention helps someone feel valuable.
(15) Finding the right person will create emotional stability.
(16) A meaningful relationship will lead to lasting happiness.
(17) A loving relationship will lead to a happily ever after.

Expected relational disappointment (ERD):

(1) Deep relationships do not fix emotional discontent.
(2) Even in a close relationship, it can be as lonely as when someone is single.
(3) Even after finding a deep relationship, there is still a longing for something more.
(4) After entering a close relationship, someone will likely feel emotionally the same.
(5) A close relationship does not erase one’s personal struggles.
(6) A close relationship will eventually end in disappointment.
(7) Closeness with someone does not solve someone’s deeper problems.
(8) People still feel unhappy when they are in love.
(9) There is a letdown by how little changes after becoming close to someone.

## Appendix B. Fornell–Larcker Criterion and HTMT Results

**Table A1:** Fornell–Larcker criterion (development sample).

Construct	1	2	3	4	5
1. RFB factor 1	**0.808**				
2. RFB factor 2	0.346	**0.768**			
3. ERD	−0.412	−0.289	**0.731**		
4. Personal wellbeing	0.512	0.487	−0.268	**0.805**	
5. Unrealistic expectations	0.301	0.428	−0.221	0.119	**0.663**

Notes: RFB = rescue fantasy beliefs. ERD = expected relational disappointment. Diagonal elements (in bold) represent the square root of average variance extracted (AVE). Off-diagonal elements represent inter-construct correlations.

**Table A2:** Fornell–Larcker criterion (validation sample).

Construct	1	2	3	4	5
1. RFB factor 1	**0.827**				
2. RFB factor 2	0.362	**0.716**			
3. ERD	−0.443	−0.312	**0.708**		
4. Personal wellbeing	0.441	0.402	−0.201	**0.753**	
5. Unrealistic expectations	0.278	0.356	−0.184	0.098	**0.586**

Notes: RFB = rescue fantasy beliefs. ERD = expected relational disappointment. Diagonal elements (in bold) represent the square root of average variance extracted (AVE). Off-diagonal elements represent inter-construct correlations.

**Table A3:** HTMT ratios (development and validation samples).

Construct Pair	Development Sample	Validation Sample
RFB factor 1–RFB factor 2	0.590	0.550
RFB factor 1–ERD	0.630	0.600
RFB factor 1–personal wellbeing	0.660	0.580
RFB factor 1–unrealistic expectations	0.480	0.460
RFB factor 2–ERD	0.520	0.500
RFB factor 2–personal wellbeing	0.570	0.530
RFB factor 2–unrealistic expectations	0.610	0.580
ERD–personal wellbeing	0.440	0.420
ERD–unrealistic expectations	0.390	0.360
Personal wellbeing–unrealistic expectations	0.410	0.380

Notes: RFB = rescue fantasy beliefs. ERD = expected relational disappointment. HTMT = heterotrait–monotrait ratio of correlations. Values below 0.85 indicate adequate discriminant validity (Henseler et al., 2015).

## Appendix C. Table of Scales and Items

**Item Number**	**Scales and Items**
	Rescue fantasy beliefs (RFB) scales (six items and two factors)
	Factor 1: Relational support expectancy
(1)	The right relationship will end everything wrong in someone’s life
(2)	A fulfilling relationship will end the need for much else
(3)	A charming prince or princess in someone’s life will save them from life’s challenges
	Factor 2: Relational self-worth enhancement
(1)	A close relationship positively lifts how someone views themselves
(2)	A close relationship will help someone feel better about themselves
(3)	Relationship attention helps someone feel valuable
	Expected relational disappointment (ERD)
(1)	Deep relationships do not fix emotional discontent
(2)	Even in a close relationship, it can be as lonely as when someone is single
(3)	A close relationship does not erase one’s personal struggles
	Current relational satisfaction Rate how dissatisfied/satisfied you are with your close relationship(s) on the following qualities
(1)	Positivity
(2)	Safety
(3)	Adventure
(4)	Trust
	Shopping addiction (Andreassen et al., 2015)
(1)	I think about shopping/buying things all the time
(2)	I shop/buy things in order to change my mood
(3)	I shop/buy so much that it negatively affects my daily obligations (e.g., school and work)
(4)	I feel I have to shop/buy more and more to obtain the same satisfaction as before
(5)	I have decided to shop/buy less, but have not been able to do so
(6)	I feel bad if I, for some reason, am prevented from shopping/buying things
(7)	I shop/buy so much that it has impaired my wellbeing
	Gender (female)
(1)	What is your gender? 1—male, 2—female, 3—nonbinary
	Hours on social media (weekly average)
(1)	On average, how many hours are you on social media each week? 0, 1, 2, 3, 4, 5, 6, 7, 8, 9, 10, 11+
Notes: There was a random presentation of items within each scale section. Participants indicated their current relationship status by selecting one option from a predefined list of categories. Options included: (1) single (not in a relationship and not dating), (2) dating casually (seeing one or more people casually), (3) dating seriously (seeing one or more people with the intent of a long-term relationship), (4) in an open or polyamorous relationship (having multiple partners with the consent and knowledge of all involved), (5) married or domestic partnership, (6) divorced (legally divorced and not remarried), (7) cohabiting (living together with a partner in a committed relationship but not married), (8) in an exclusive relationship (in a committed, monogamous relationship but not married or cohabiting), (9) separated (legally married but no longer living with spouse), (10) widowed (a spouse has passed away and not remarried), and (11) other [fill in blank]. Participants who selected (1) single (not in a relationship and not dating) were dummy coded as 1, with all other options coded as 0. Body mass index (BMI) was calculated from self-reported height (feet/inches) and weight (pounds) information.	
